# Prediction of the dose range for adverse neurological effects of amiodarone in patients from an in vitro toxicity test by in vitro–in vivo extrapolation

**DOI:** 10.1007/s00204-021-02989-2

**Published:** 2021-02-19

**Authors:** Engi Abd el-Hady Algharably, Emma Di Consiglio, Emanuela Testai, Reinhold Kreutz, Ursula Gundert-Remy

**Affiliations:** 1 Institute of Clinical Pharmacology and Toxicology, Charité-Universitätsmedizin Berlin, Corporate Member of Freie Universität Berlin, Humboldt-Universität Zu Berlin, Berlin Institute of Health, Berlin, Germany; 2 Istituto Superiore Di Sanità, Environment and Health Department, Mechanisms, Biomarkers and Models Unit, Rome, Italy; 3 DZHK (German Centre for Cardiovascular Research), partner site Berlin, 10115 Berlin, Germany

**Keywords:** Animal alternative, Dose–response modeling, In silico, Neurotoxicity, Physiologically based pharmacokinetic modeling, Reverse dosimetry

## Abstract

Amiodarone is an antiarrhythmic agent inducing adverse effects on the nervous system, among others. We applied physiologically based pharmacokinetic (PBPK) modeling combined with benchmark dose modeling to predict, based on published in vitro data, the in vivo dose of amiodarone which may lead to adverse neurological effects in patients. We performed in vitro–in vivo extrapolation (IVIVE) from concentrations measured in the cell lysate of a rat brain 3D cell model using a validated human PBPK model. Among the observed in vitro effects, inhibition of choline acetyl transferase (ChAT) was selected as a marker for neurotoxicity. By reverse dosimetry, we transformed the in vitro concentration–effect relationship into in vivo effective human doses, using the calculated in vitro area under the curve (AUC) of amiodarone as the pharmacokinetic metric. The upper benchmark dose (BMDU) was calculated and compared with clinical doses eliciting neurological adverse effects in patients. The AUCs in the in vitro brain cell culture after 14-day repeated dosing of nominal concentration equal to 1.25 and 2.5 µM amiodarone were 1.00 and 1.99 µg*h/mL, respectively. The BMDU was 385.4 mg for intravenous converted to 593 mg for oral application using the bioavailability factor of 0.65 as reported in the literature. The predicted dose compares well with neurotoxic doses in patients supporting the hypothesis that impaired ChAT activity may be related to the molecular/cellular mechanisms of amiodarone neurotoxicity. Our study shows that predicting effects from in vitro data together with IVIVE can be used at the initial stage for the evaluation of potential adverse drug reactions and safety assessment in humans.

**Supplementary Information:**

The online version contains supplementary material available at 10.1007/s00204-021-02989-2.

## Introduction

In vitro toxicity assays emerge as an appealing alternative to animal-based toxicity testing aiming to decrease the reliance on animal experimental studies performed in quantitative risk assessment of drugs and chemicals (Adler et al. 2011; Punt et al. 2018; Strikwold et al. 2013). They offer the advantages of reduced cost and time, and are in accordance with the aim to replace and reduce the use of animals in toxicological testing (Lilienblum et al. 2008; Punt et al. 2018). Nevertheless, the implementation of in vitro assays in quantitative risk assessment is still limited, mainly by its inability to adequately mimic the complex biological and cellular interactions in the in vivo environment (Lilienblum et al. 2008; Zhang et al. 2018b). Furthermore, in vitro assays alone cannot directly provide in vivo dose–response relationship from which a point of departure can be derived for risk assessment purposes. In vitro data capable of elucidating mechanisms of toxic effects can thus be combined with physiologically based pharmacokinetic (PBPK) modeling applying reverse dosimetry or in vitro*–*in vivo extrapolation (IVIVE). This approach has been recognized as a useful tool to evaluate the chemical safety of substances (Sewell et al. 2017). PBPK models can predict blood or tissue concentrations of a compound or its metabolite(s) over time at any dose, and in combination with concentration-effect data, they allow the analysis of various dosing scenarios. This approach enables the translation of in vitro concentration–response relationships into in vivo dose–response curves that are used to define safe exposure levels in an organism (Louisse et al. 2017; Zhang et al. 2018a).

Amiodarone is a potent antiarrhythmic drug effective against both atrial and ventricular arrhythmias (Auer et al. 2002; Williams and Viswanathan 2013). However, because of a wide spectrum of adverse effects involving the heart, the lungs, liver and CNS, its use has to be carefully evaluated and is currently restricted to the management of serious ventricular arrhythmias (Priori et al. 2015; Vassallo and Trohman 2007). The reported incidence of neurological adverse effects, induced by amiodarone, in the literature ranges from 2.8 to 74% (Greene et al. 1983; Hilleman et al. 1998; Orr and Ahlskog 2009). The assessment of the neurotoxic potential of chemicals using animal studies is not only laborious but also challenging in terms of the difficulty of interpreting the observed functional changes and linking it directly to the chemical and not to indirect hormonal or immunological stimuli (Harry and Tiffany-Castiglioni 2005). In the same way, the relevance of in vitro neurotoxicity assay models could be enhanced if they are designed to closely mimic the complex nature of the brain, providing reliable information regarding the ability for drugs/chemicals to cross-cellular barriers and interact with cell systems (Bokhari et al. 2007; Schmidt et al. 2017). In a previous work, the in vitro neurotoxicity and the biokinetic profile of amiodarone in a rat brain cell model were investigated at clinically relevant amiodarone concentrations that have been reported to be associated with neurotoxicity in patients (Lafuente-Lafuente et al. 2009; Pomponio et al. 2015b).

The aim of the current study was to apply a PBPK modeling-based reverse dosimetry approach for extrapolation from in vitro results to the clinical situation using a human adapted PBPK model to translate intracellular concentration–time data from rat brain cells into a human in vivo dose. In addition, we used the predicted human doses to construct a human in vivo dose–response relationship based on the in vitro pharmacodynamic response. By this, we could demonstrate the potential offered using IVIVE and the predictive value of using in vitro data.

Furthermore, by doing so, we investigate the applicability of the chosen toxicological endpoint as a valid marker to predict amiodarone neurotoxicity in the brain.

## Methods

### Acquirement of in vitro concentration–response data in a rat brain model

We used available data on amiodarone in vitro biokinetic in a rat brain cellular model previously published (Pomponio et al. 2015b). A step-wise procedure aimed to complement in vitro toxicity testing of test compounds with biokinetic assessment was established within an EU funded Project (Predict IV) (https://cordis.europa.eu/project/rcn/86700en.html). In this study, 3D re-aggregating brain cells were repeatedly treated every other day for 14 days with amiodarone at two concentration levels: 1.25 and 2.5 µM. The biokinetic profile of amiodarone in the cell culture was followed for 24 h on the first (day 0) and the last (day 14) day of treatment. Along the 24 h, five time points were selected to measure the actual concentration of the parent compound (amiodarone) and its main metabolite mono-N-desethylamiodarone (MDEA), in all the compartments (medium, cells and plastic device). In the same study, the choline acetyl transferase (ChAT) activity was used as one of the neurotoxicity markers after 14-day repeated exposure of the in vitro rat brain model to amiodarone. The progress of the neurotoxic insult was then compared with the kinetics of amiodarone. To perform the PBPK modeling, the measured in vitro concentrations, which were expressed as concentrations per flask in the publication, were recalculated as concentrations in µg/mL.

### PBPK model

**Fig. 1:**
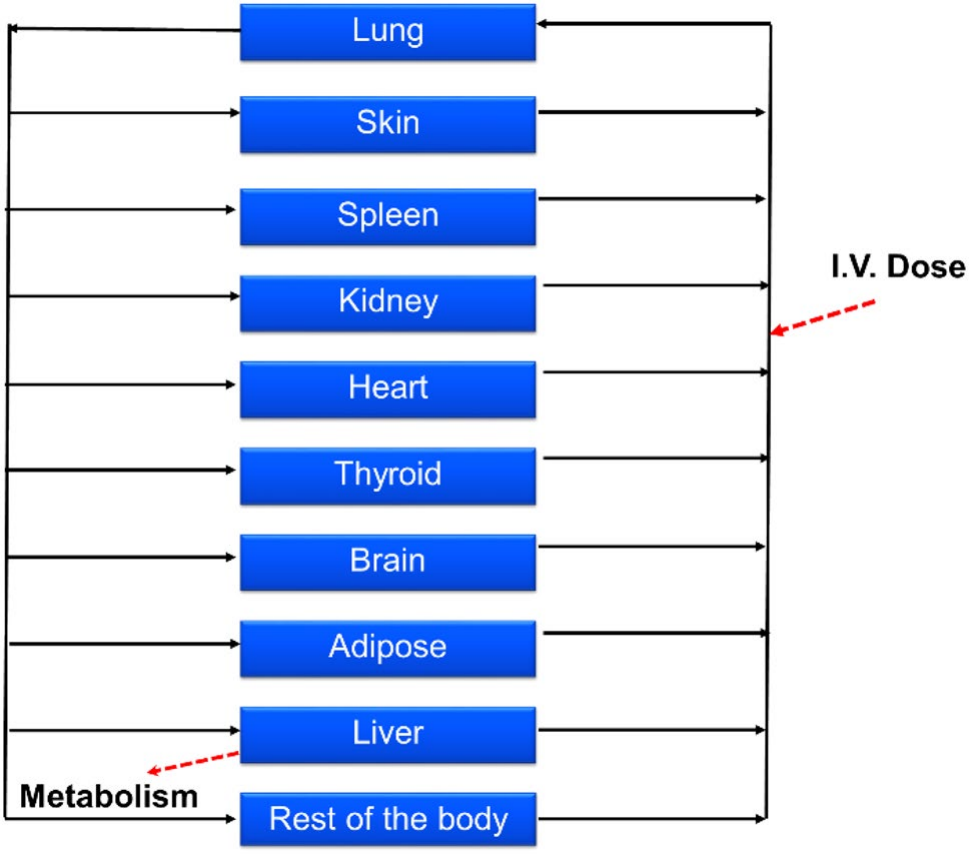
Whole-body PBPK model structure with drug input by the i.v. route

We used a validated rat kinetic model that has been previously developed using data from experimental studies in rats (Lu et al. 2016) and applied to humans (Algharably et al. 2019) giving simulations that were in good agreement with the in vivo observed amiodarone time-courses in patients after intravenous (i.v.) application published in the literature. Briefly, the model consisted of 10 tissue compartments as well as arterial and venous blood, and the circulation system is closed via the lung and the heart. The input of amiodarone was modeled by the i.v. route (Fig. 1) since the model using this route of administration was validated with experimental data from patients in our previous work (Algharably et al. 2019). Drug distribution into tissues was modeled as permeability rate-limited kinetics, where the transport of drug molecules between the two sub-compartments is described by factors such as the permeability–surface area product (PS × tissue) and the tissue-specific unbound fraction of amiodarone as described before (Algharably et al. 2019; Lu et al. 2016). Amiodarone excretion was modeled by metabolism in the liver via constant clearance to give MDEA as a primary metabolite as shown in vivo (Ha et al. 2005) and in in vitro with different hepatic cellular models (Pomponio et al. 2015a). Other tissue compartments were regarded as non-metabolizing; this assumption was extended to the brain since, although some cytochrome P450 enzymes are present in the brain, their expression levels are generally low as it was demonstrated also in the 3D cellular model used as source of in vitro data (Vichi et al. 2015). As a consequence, the metabolic activity in the brain does not significantly contribute to the overall body clearance (McMillan and Tyndale 2018; Woodland et al. 2008). This also becomes evident when comparing the molar concentration of MDEA formed in the cell lysate relative to amiodarone concentration after exposure between the in vitro primary human hepatocyte culture (Pomponio et al. 2015a) and brain 3D cell culture (Pomponio et al. 2015b). The rate of MDEA formation was about 2.5–3% versus 50–60% in the brain cells and liver cells, respectively. The model output was once again tested and compared to the published experimental data in rat (Riva et al. 1982; Shayeganpour et al. 2008; Wyss et al. 1990).

The area under the curve (AUC) of the mean amiodarone concentrations in rat brain cell lysate on dosing day 14 was selected as the kinetic metric for the IVIVE. For optimization of the dose, we iterated the doses in steps of 0.01 mg amiodarone given into human system and selected the dose which had the smallest difference between the predicted and the measured value. In an alternative approach, we performed the same process applying the observed *C*_max_ as well as using the nominal concentrations of the in vitro experiment. For simulation, we used Berkeley Madonna software (version 8.3.18).

### Prediction of in vivo dose–response using the PBPK model-facilitated reverse dosimetry approach

We used AUC based on 24 h period, i.e. AUC_0–24_, on day 14 after repeated exposure to obtain corresponding in vivo repeated doses in the human by using the PBPK model. In the next steps, in vivo doses were calculated for the in vitro dosing in the publication of Pomponio et al. (2015a, b) and related to the toxic effect observed in vitro. By performing this exercise, in vitro concentration–response data were translated into in vivo dose–response data in the human for ChAT activity. The same steps were performed for predictions based on *C*_max_.

### Benchmark dose (BMD) analysis of predicted in vivo dose–response data

BMD modeling was applied on the predicted in vivo dose–response human data using the R-package PROAST (version 67.0) (www.proastweb.rivm.nl). Models for continuous data were used and a benchmark response (BMR) was set at an effect size of 1 standard deviation (SD) of the background response for ChAT activity (EFSA Scientific Committee et al. 2017; Slob 2017). A 90% confidence interval around the BMD (dose giving BMR) for reduced ChAT activity was estimated with a lower bound (BMDL) and upper bound (BMDU). The goodness of fit application of the models was used to determine if the model could be accepted with *P* > 0.05. All models which met the requirements for acceptance of the model fit were considered by model averaging to derive a single BMD confidence interval from the set of BMD confidence intervals for the chosen neurotoxicity endpoint (Supplementary Table S1, S2, Fig. S1). We selected BMDU to predict a dose able to cause adverse effects on the CNS which will be compared with doses associated with neurotoxicity from clinical studies. In a last step, the dose, which was obtained using an intravenous input into the model, was converted into oral doses using an oral bioavailability factor of 0.65 as described in the literature (Pourbaix et al. 1985).

### Alternative approach using the nominal concentrations

To compare predictions based on measured concentrations in cell lysate, which represent intracellular concentrations, we performed also the same process starting with the nominal concentrations. In this approach, it has to be taken into consideration that the nominal concentration in a protein free medium (Pomponio et al. 2015a, b) corresponds to the concentration of the free fraction of amiodarone. Therefore, the resulting estimates must be corrected for protein binding (*F*_u_ = 0.06).

### Use of rat data

We performed the same process also using the PK rat model for the kinetic data and calculated the BMDU using the pharmacodynamics data of the in vitro model.

### Relevance of PBPK model-based predictions

To assess the predictive value of the modeling approach, the dose obtained for humans, corresponding to the upper limit of BMD, was compared to amiodarone doses that elicited adverse reactions in the nervous system in clinical practice. Because in clinical long-term treatment amiodarone is used orally, we calculated the corresponding oral dose by applying a bioavailability of 65% after oral administration (Pourbaix et al. 1985).

## Results

### Human PBPK modeling-reverse dosimetry based on AUC and ***C***_max_ from the brain cell lysate

**Fig. 2:**
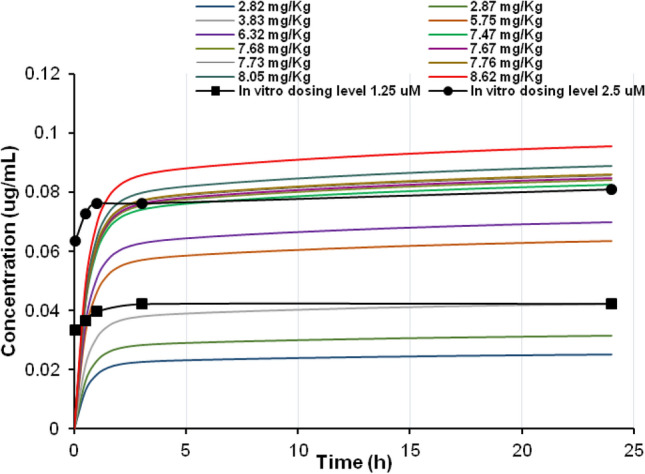
Optimization of the intravenous amiodarone dose to simulate the in vivo human intracellular concentration in brain (*lines*) as close as possible to the in vitro intracellular concentrations data in rat brain measured over 24 h on day 14 of repeated exposure at two dosing levels (*closed circles* and *squares*) (Pomponio et al. 2015b)

**Fig. 3:**
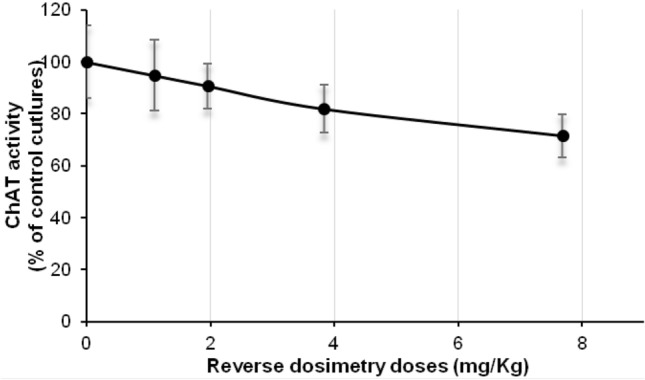
Predicted dose–response curve for amiodarone effect on ChAT activity in human brain cells based on AUC as kinetic metric

The AUCs resulting from the concentrations measured in the in vitro brain cell lysate after daily repeated exposure to 1.25 and 2.5 µM amiodarone on day 14 were 1.00 µg*h/mL and 1.99 µg*h/mL, respectively, and the doses calculated by IVIVE were 3.83 and 7.68 mg/kg, respectively (Table 1, Fig. 2). Figure 3 shows the in vivo dose–response curves for the decline in ChAT activity, whereby the in vivo doses were predicted from the in vitro concentrations using the human PBPK model. On the other hand, the doses calculated by IVIVE for the observed in vitro *C*_max_ of 0.042 and 0.081 µg/mL that correspond to 1.25 and 2.5 µM level of exposure after 14 days were 3.76 and 7.12 mg/kg, respectively (Supplementary Fig. S2).

**Table 1:** AUC in rat brain cell culture (data taken from Pomponio et al. (2015b) and in vivo human dose obtained by reverse dosimetry to simulate the in vitro AUC

Concentration of daily dosing (μM)	AUC (μg*h/mL)	Dose resulting from reverse dosimetry (mg/kg)
1.25	1.00	3.83
2.5	1.99	7.68

### BMD analysis of predicted dose–response data, and evaluation of the predictive value of the approach

BMD modeling was applied on the predicted in vivo dose–response data in human to determine BMDU and BMDL value for ChAT activity. The predicted human BMDU and BMDL were 5.28 and 1.3 mg/kg i.v., respectively, based on the AUC approach and were 5.09 and 1.32 mg/kg i.v., respectively, based on the *C*_max_ approach (Table 2).

**Table 2:** BMD confidence interval and total dose predicted for human based on AUC, *C*_max_ and nominal concentration as kinetic metric

Approach	BMD_lower_ (mg/kg)	BMD_upper_ (mg/kg)	Total oral dose in human (mg)
AUC	1.3	5.28	593
*C*_max_	1.32	5.09	571.6
Nominal concentration	0.012	0.058	10833.3^a^

BMD modeling was based on model averaging

^a^The nominal concentrations are regarded as free drug concentration in the in vitro model. The resulting BMDU (6.5 mg orally) is converted to the corresponding total dose after multiplying by 100/unbound fraction (*F*_u_ = 0.06)

### Evaluation of the predicted dose for human adverse neurological effects

As the predicted doses (BMDU, BMDL) were based on an i.v. model and clinical data on neurological toxicity are only reported from studies with oral administration of amiodarone, we calculated the corresponding oral dose for the BMDU. The oral dose for the BMDU would be 8.12 mg/kg bw or 593 mg for a standard human subject (73 kg) with the AUC approach and 7.83 mg/kg bw or 572 mg for the *C*_max_ approach (Table 2).

When starting the modeling using nominal concentrations the resulting dose was 10,833 mg considering that the nominal concentration corresponds to the free fraction.

In the literature, doses of 400–600 mg were reported to be associated with neurological toxicity in clinical studies (Kerin et al. 1989; Orr and Ahlskog 2009; Smith et al. 1986). The spectrum of symptoms reported included muscle weakness, fatigue, tremor, ataxia, peripheral neuropathy, and cognitive impairment.

Regarding the rat PBPK-BMD modeling we obtained a BMDU of 17 mg/kg i.v. corresponding to 48.58 mg/kg orally by applying an oral bioavailability factor (*F*) of 0.35 (Shayeganpour et al. 2005) (data not shown). In the literature, doses of 400–500 mg/kg orally were reported to be associated with neurological toxicity (Costa-Jussa and Jacobs 1985; Vereckei et al. 1993).

## Discussion

Though animal testing is the principal experimental approach for assessing neurotoxic potential of chemicals and in preclinical studies, emerging non-animal testing integrated strategies with proposed neurotoxicity models with some predictive value are becoming available for endpoints concerning the mode of action (Colaianna et al. 2017).

The primary information which can be obtained by in vitro assays is still limited for use in risk assessment and for predicting safe clinical doses or doses potentially eliciting adverse effects. Indeed, the data coming from in vitro investigations only provide concentration–response relationship rather than the actual doses related to in vivo adverse effects, sometimes on end-points not necessarily biologically meaningful and recognized as biomarkers of in vivo adversity (Blaauboer et al. 2012). In addition, extrapolation should be based on actual cell exposure rather than on nominal concentration. To overcome these limitations, the biokinetic approach described by Kramer et al. (2015) and in the recent OECD GD GIVIMP (OECD 2018) complemented with PBPK modeling in an IVIVE approach has increasingly been used to relate (toxic internal) concentrations to external doses. This approach facilitates the quantitative description of ADME processes of a compound in the body and the target organ(s) (Rietjens et al. 2011).

The aim of the present study was to assess whether in vivo adverse effects of amiodarone on the nervous system can be predicted by combined PBPK/BMD modeling approaches, based on in vitro evaluation of both biokinetic parameters and markers of neurotoxicity.

In this work, we started form a PBPK model that was previously tested and validated to adequately describe the kinetics of amiodarone as well as its tissue concentrations in rat (Lu et al. 2016) and humans (Algharably et al. 2019). We used it to translate the in vitro data on ChAT inhibition of amiodarone and the in vitro biokinetic data in the brain cell lysate acquired from the work published by Pomponio et al. (2015b) in rat brain cells into human dose–response relationship using the IVIVE approach. In contrast to many other studies, in which nominal concentrations are reported, in this study the authors provided measured, actual concentrations over a 24-h period in the cell lysate and in the supernatant after single (first day of treatment) and multiple dosing (14th day of treatment every other day) (Pomponio et al. 2015b). Actual concentrations reflect the real exposure of cell which can greatly influence the toxicological outcome, especially after repeated exposure (Coecke et al. 2013), while nominal concentration are the theoretical concentrations of substances at the time of their preparation and use in the test. Hence, they are not measures of true exposure and do not provide dose–response data that can be employed in risk assessment (Louisse et al. 2017). This aspect has been well evidenced by the results obtained when we performed the modeling with nominal concentrations. The resulting doses, not considering processes affecting the actual exposure, e.g. adsorption of amiodarone to plastic material and/or the distribution into the cells (Table 2, supplementary Fig. S3) were, therefore, higher than the doses in clinical studies eliciting adverse effects in the CNS. In the PBPK-reverse dosimetry approach, in vitro intracellular effect concentrations are considered the most proper value as surrogate for the tissue concentrations that elicit toxicity in vivo (Louisse et al. 2017). The AUCs calculated from these data were selected as proxy for amount of amiodarone in the cell and, therefore, the appropriate metric in the target tissue given the long elimination half-life of amiodarone and the limited metabolic capacity of brain cells. In accordance with other authors, we consider the integrated concentration of the chemical in target tissue over time (i.e., AUC) a more suitable dose metric for chronic effects of compounds (Thompson et al. 2008), whereas acute toxicity is often regarded to result from high exposure in which peak concentrations (*C*_max_) are usually considered more informative (Rietjens et al. 2019). Notwithstanding, after performing the same IVIVE process coupled with BMD modeling using the observed *C*_max_ in the in vitro experiment, results were still comparable to those obtained from using the AUC metric.

By applying BMD modeling on the predicted dose–response curve we selected an effect size of 1 SD for BMR, hence, the resulting BMDL would be close to or even overlapping with a dose not yet eliciting an adverse effect. However, in our study, we were interested in predicting a dose from an in vitro study that would elicit adverse effects in vivo. In this respect, we considered that the BMDU would be more appropriate, because the BMDU represents a dose at which an adverse effect is assumed to become evident. It is worth noting that the modeling approach should also conform with the dosing regimen used in clinical practice. Prediction for BMDU for single dose exposure is not comparable to the clinical scenario of multiple dosing in patients since amiodarone is typically given for prolonged periods to control chronic conditions such as arrhythmias. It is not surprising that AUC_0-24_ after single dose exposure were lower compared to those after 14 days of repeated exposure (AUC_0-24_ = 0.007 and 0.0144 μg/mL*h for 1.25 and 2.5 µM, respectively, after single exposure vs. AUC_0-24_ = 1.00 and 1.99 µg*h/mL, for 1.25 and 2.5 µM, respectively, after repeated exposure). The latter is a strength offered by the in vitro cell model that we used that emulate a multiple dosing clinical scenario. As such, information on repeated cellular exposure is not often available.

Using a validated i.v. human amiodarone model for reverse dosimetry, we calculated the BMDU of amiodarone for the endpoint ChAT inhibition as 5.28 mg/kg bw for an i.v. dose corresponding to 8.12 mg/kg bw and 593 mg per person for an oral dose. Performing a literature search, we identified several reports with neurological adverse effects caused by amiodarone. In these studies, the most common neurotoxicity findings included tremor, ataxia and peripheral neuropathy (Ishida et al. 2010; Kang et al. 2007; Orr and Ahlskog 2009; Palakurthy et al. 1987). Adverse effects such as muscle weakness, fatigue, tremor, ataxia, peripheral neuropathy, and cognitive impairment/encephalopathy have been reported to occur with amiodarone treatment (Harris et al. 1983; Palakurthy et al. 1987) and clinically significant neurotoxic effects were observed when relatively high doses (400–600 mg) have been used in clinical practice before (Orr and Ahlskog 2009). The outcome of the in vivo human dose–response modeling resulted in BMDU of 593 mg based on AUC metric and 572 mg based on *C*_max_ metric. Such doses are in excellent agreement with the doses of 400–600 mg which are reported to be associated with neurological toxicity in clinical studies (Kerin et al. 1989; Orr and Ahlskog 2009; Smith et al. 1986). In contrast, dose prediction based on the nominal concentrations resulted in a BMDU of 10,833.3 mg orally (Table 2 and supplementary Fig. S3) which is clearly higher than the clinical doses eliciting adverse effects and demonstrates the inadequacy of using the nominal concentrations to predict toxicity in the in vivo situation.

A few studies in the literature addressed amiodarone neurotoxicity in rats (Costa-Jussa and Jacobs 1985; Rao et al. 1986; Vereckei et al. 1993; Yamanaka et al. 2019) where doses ranging from 400 to 500 mg/kg orally (Costa-Jussa and Jacobs 1985; Vereckei et al. 1993) were reported to be associated with severe neurological toxicity in all animals. However, all these doses were selected to elicit neurotoxicity and in none of the studies, no observed adverse effect level (NOAEL) was reported. Hence, as the dose in the range between overt toxicity and no toxicity is not known a comparison between the obtained BMDU of 48.58 mg/kg orally and the observed frank neurotoxic doses is not suitable for challenging the selected endpoint for neurotoxicity as being a predictive surrogate endpoint.

The underlying mechanism for the observed adverse neurological effects is not yet fully elucidated (Orr and Ahlskog 2009; Palakurthy et al. 1987). Some authors propose that amiodarone can bind to phospholipids, as it has been observed in hepatic cells, where it induces phospholipidosis (Pomponio et al. 2015a) or that the accumulation of lipids within lysosomes in neuronal cells and their processes may be involved (Costa-Jussa and Jacobs 1985). A characteristic finding in amiodarone-induced neuropathy in humans was the presence of lysosomal inclusions in all cell types in the nerves (Costa-Jussa and Jacobs 1985; Jacobs and Costa-Jussa 1985). Cytoplasmic changes were observed in Schwann cells of myelinated and unmyelinated axons, involving the loss of the organelles with a consequence in the myelin sheath breakdown (Jacobs and Costa-Jussa 1985). These latter changes are in line with the observed inhibitory effects of amiodarone in animal studies on lysosomal phospholipases A1 and A2 (Heath et al. 1985) with large accumulations of lysosomal bodies in nerve cells and processes and evidence of degenerative changes (Costa-Jussa and Jacobs 1985; Rao et al. 1986). Although the used in vitro data were obtained from rat cells, these pathological findings suggest to a large extent a similarity between rat and human in the toxicodynamics of amiodarone regarding neurotoxicity. An alternative to the effects on lysosomal damage might be offered by the finding that amiodarone as well as its primary metabolite MDEA bind to calmodulin. Thus, inhibiting calmodulin stimulation of phosphodiesterase and synaptic membrane Ca^2+^-ATPase (Kodavanti et al. 1992) during treatment with amiodarone may lead to perturbed Ca^2+^ homeostasis and sustained increase in [Ca^2+^] associated with cell injury (Kodavanti et al. 1992). Inhibition of ChAT activity in a calcium-dependent manner has been described for veratridine, a known neurotoxic agent (Loureiro-Dos-Santos et al. 2001). Hence, perturbation of Ca^2+^ homeostasis may offer an explanation for impaired ChAT activity after exposure to amiodarone. Inhibition or loss of function of ChAT which is the enzyme utilizing acetyl-CoA for choline acetylation to catalyse the synthesis of acetylcholine has been proposed as a mechanism of neurotoxicity of phenylacetyl-CoA (Potempska et al. 1984), aluminium (Bilkei-Gorzo 1993), amyloid-beta (Nunes-Tavares et al. 2012), and toxic agents such as AF64A (Mantione et al. 1983), quinolinic acid (Boegman et al. 1985), and ethylcholine mustard aziridinium (ECMA) (Pillar et al. 1988). Symptoms of amiodarone neurotoxicity include in addition to peripheral neurotoxicity, central symptoms that could be explained by cholinergic dysfunction aspects including encephalopathies and cognitive impairment bearing resemblance to neurodegenerative diseases such as Alzheimer’s disease where loss of cholinergic neurons and a decrease in ChAT enzyme activity occur in cholinergic areas of the diseased brains (Pappas et al. 2000). Taken together, the inhibition of ChAT might explain mechanistically amiodarone neurotoxicity and a relevant mode of action.

To exert its effect in the CNS, permeating the blood brain barrier (BBB) is crucial for amiodarone. This has been reported in vivo in rats (Riva et al. 1982) and in in vitro system from animal tissues (Schultz et al. 2015). Such information is not directly available in humans, but due to the lipophilicity of the drug, the passive passage through the BBB is expected as well. Passage of the BBB is an important aspect that should be considered in conjunction with the biokinetic behavior of the drug in the assessment of neurotoxicity since it affects the ability of the in vitro test to predict neurotoxic doses from target tissue levels (Forsby and Blaauboer 2007). In our model, parameters describing drug partitioning between blood and tissues including the brain were incorporated in the model, the output of which was validated as it was compared to experimental rat studies reporting amiodarone concentrations in the brain (Riva et al. 1982; Shayeganpour et al. 2008; Wyss et al. 1990). Hence, crossing the BBB was captured in the model, also in quantitative terms. The in vitro rat brain 3D culture model did not incorporate a BBB. However, given the physicochemical properties of amiodarone omitting the BBB in the model can be assumed not to influence the in vivo distribution to a relevant extent.

Overall, the conformity of our prediction and the findings on adverse effects in clinical studies may be seen as supporting the hypothesis that impaired ChAT activity is related to the molecular/cellular mechanisms involved in amiodarone neurotoxicity.

Moreover, the metabolite MDEA is known to be pharmacologically active (Kato et al. 1988) and on the basis of some in vitro results, it has been suggested that it can contribute to the hepatotoxicity of amiodarone treatment (Zahno et al. 2011). Since brain cells are endowed with a limited metabolic capacity in forming MDEA (Pomponio et al. 2015b), a role of in situ metabolism in the onset of neurological effects could not be excluded in principle.

In conclusion, our study shows the value of PBPK modeling and IVIVE for predicting adverse drug reactions from in vitro toxicodynamic data and indicate that it can contribute to support hypothesis on the mechanism of action.

## Supplementary Information

Below is the link to the electronic supplementary material.

Supplementary file1 (DOCX 75 KB)

## Data Availability

NA.

## Code availability (software application or custom code)

Berkeley Madonna software (version 8.3.18).
