# Supplementary material for: Prediction of the dose range for adverse neurological effects of amiodarone in patients from an in vitro toxicity test by in vitro–in vivo extrapolation
Source: Arch Toxicol. 2021 Feb 19;95(4):1433–42. doi: 10.1007/s00204-021-02989-2 (PMC8032623; doi:10.1007/s00204-021-02989-2)
Supplement: Supplementary file 1 — Supplementary file1 (DOCX 75 KB) [file 204_2021_2989_MOESM1_ESM.docx]

**Supplementary file**


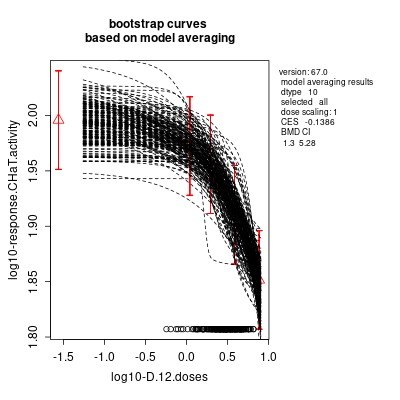


**Fig. S1 BMD modeling for the dose-response curve of amiodarone effect on ChAT activity in human based on AUC approach.** BMR= 0.1386, model averaging, number of bootstrap runs =200, AIC criterion 2.

**Table S1 Fitted models for the dose-response curve of amiodarone effect on ChAT activity in human based on AUC as kinetic metric**

| **Model** | **Converged** | **Loglik** | **Npar** | **AIC** |
| --- | --- | --- | --- | --- |
| full model | 1 | 23.34 | 6 | -34.68 |
| null model | 1 | 12.04 | 2 | -20.08 |
| Expon. m3- | 1 | 23.26 | 4 | -38.52 |
| Expon. m5- | 1 | 23.33 | 5 | -36.66 |
| Hill m3- | 1 | 23.26 | 4 | -38.52 |
| Hill m5- | 1 | 23.33 | 5 | -36.66 |
| Inv.Expon. m3- | 1 | 23.30 | 4 | -38.60 |
| Inv.Expon. m5- | 1 | 23.32 | 5 | -36.64 |
| LN m3- | 1 | 23.29 | 4 | -38.58 |
| LN m5- | 1 | 23.32 | 5 | -36.64 |

**Table S2 Model weights**

| **Model** | **Weight** |
| --- | --- |
| EXP | 0.2456 |
| HILL | 0.2456 |
| INVEXP | 0.2556 |
| LOGN | 0.2531 |

**Fig. S2** Optimization of the intravenous amiodarone dose based on C_max_ approach to simulate the *in vivo* human intracellular concentration in brain (lines) as close as possible to the *in vitro* intracellular concentrations data in rat brain at two dosing levels (closed circles and squares) (Pomponio et al. 2015b)

2.5uM 1. 25 uM 0.625 uM 0.325 uM

1

**Fig. S3** Optimization of the intravenous amiodarone dose to simulate the *in vivo* plasma concentration (lines) as close as possible to the four *in vitro* nominal concentrations (closed symbols) (Pomponio et al. 2015b).
